# Access to clean energy in Africa revisited: The roles of women empowerment, corruption control, FDI and sectoral growth

**DOI:** 10.1371/journal.pone.0317781

**Published:** 2025-02-04

**Authors:** Kwame Adjei-Mantey, Paul Adjei Kwakwa, Eleazer Ankrah

**Affiliations:** 1 Institute of Statistical, Social and Economic Research, University of Ghana, Accra, Ghana; 2 Department of Entrepreneurship and Business Science, University of Energy and Natural Resources, Sunyani, Ghana; 3 School of Sustainable Development, University of Environment and Sustainable Development, PMB Somanya, Ghana; Obafemi Awolowo University, NIGERIA

## Abstract

One of the key contributors to climate change is energy consumption, with the type of energy used having implications on the natural environment and health of users. To promote environmental sustainability and sustainable development, Sustainable Development Goal (SDG) 7 aims to achieve accessibility, and affordability of clean and modern forms of energy for all. This study aims to investigate the effects of women empowerment, corruption control, foreign direct investment, and sectoral growth on access to clean energy in Africa, as well as the effects of the interrelatedness of these factors on clean energy access. Using data on 32 countries in Africa from 2002 to 2021 and rigorous econometric techniques, the study finds that women empowerment and corruption control significantly increase access to clean energy in Africa while sectoral analyses show varying effects of growth in the different sectors on clean energy accessibility. Furthermore, it is found that corruption control is not able to reverse situations of adverse effects of some variables on access to clean energy in some cases, likely due to the low levels of corruption control in Africa. The results suggest that African countries could enhance access to clean energy for its citizens and harness the full potential of clean energy, to promote sustainable development and improve the lives of their population, by empowering women, fighting corruption, and cultivating balanced economic growth.

## 1. Introduction

Climate change and the goal of maintaining a low carbon economy have dominated discussions on sustainability across the globe. This is partly because climate change has adverse implications for livelihoods while posing a threat to the sustainability of natural resources for future generations, and working towards a low carbon economy is one of the ways to slow down the rate of emissions, which contribute to climate change and consequently, environmental decline. One of the key contributors to emissions is energy use. Energy is an important input into the production of goods and services not only at the commercial and industrial level, but also at the household level. In fact, household activities including cooking and heating using solid fuels, which are often heavily polluting, contribute about a fifth of world encompassing particulate substance and a quarter of black carbon discharges, which are part of particulate matter, PM_2.5_ [1]. While clean energy use reduces environmental pollution [2–3], the use of heavily polluting fuels such as biomass fuels on the other hand pose risks to both the environment and human health [4–5]. One way out of this challenge, is to increase access to, and use of cleaner and modern energy sources particularly in developing countries, where the use of traditional and heavily polluting fuels dominates [6]. To this end, research and policy have sought to understand the factors that contribute to access to clean energy to enhance the understanding of clean energy adoption and to promote same across the developing world. Furthermore, besides the positive effects on the environment, clean energy use potentially leads to better health and economic outcomes.

Across the African region, significant disparities exist in clean energy access which demands attention. Previous research has found micro- and macro- level factors that drive clean energy accessibility in Africa. Some of the factors that have emerged to influence clean energy access in Africa include income, employment, Foreign Direct Investment (FDI), institutional quality and stable political regimes [7–10]. While the findings point to important factors, there are more factors that need to be uncovered in an attempt to explain clean energy accessibility in Africa. This study revisits the subject and this time, investigates the roles of women empowerment, corruption control and sectoral growth on access to clean energy in Africa. The study makes at least three contributions to the existing literature. Firstly, while using clean energy has been observed to potentially increase women empowerment in some cases [11–12], the reverse, which the current study seeks to explore, has received less attention; i.e., the extent to which women empowerment leads to better access to and use of clean energy is under explored. Studies such as Adusah-Poku et al. [13] have observed that if females had characteristics of males, they would have higher likelihoods of choosing cleaner cooking fuels for their households. However, whether increases in women empowerment does same is not clear and this study fills that gap. Furthermore, Masenya [14] posits that globally, women encounter restrictions that hinder their ability to contribute to productivity, welfare, decision-making, development, and crucially, their own empowerment. The results of this study will confirm if actions taken to remove such restrictions can contribute to the global efforts to reduce restricted access to clean energy. This contribution is especially important because gender gaps for women still remain wide despite the progress made over time, justifying the increasing advocacy in support of upholding women’s rights and promoting equal opportunities for women in respect of economic, social, and cultural factors. For example, in respect of economic opportunities, women earn 77 cents for every dollar paid to men and in 92 economies, there are no provisions that make equal pay for same value work mandatory [15]. There are rules that restrict women from working at night in 20 economies while in 45 economies, women are restricted from working in jobs considered dangerous [15]. These statistics suggest that the subject of promoting women empowerment is still relevant and this study will establish whether promoting women empowerment will have subsequent impacts on clean energy use. Secondly, previous findings on the role of institutional quality and political stability raises attendant questions of whether controlling corruption could have significant effects on the clean energy agenda thus prompting its investigation in this study. A good number of the countries in Africa usually score very highly in corruption and poorly in corruption control (see: 2023 Corruption Perceptions Index: Corruption… - Transparency.org). The levels of corruption have been associated with failure of governments to provide essential services and infrastructure to promote decent living since public funds are diverted through corruption into private pockets, leaving little resources available to pursue economic and social development. Controlling corruption could free up funds, providing better fiscal space for governments to make decisive interventions that would accelerate access to clean energy. The current study, thus makes a contribution by examining how corruption control potentially influences access to clean energy. More importantly the study unearths how corruption moderates the effect of FDI on access to clean energy. African countries, for many years, have attracted FDI whose effect on economic growth has been found to be positive [16]. However, little is known about its effect on access to clean energy [7]. Appealing to the pollution haven hypothesis and the pollution halo hypothesis, FDI’s effect on access to clean energy can be negative and positive respectively in developing countries such as those in Africa [17–20]. Thus, on one hand, FDI may not improve access to clean energy if the business operations resulting from the inflows do not support actions that promote clean environment; hence investors may not invest in sectors that facilitate clean energy production and accessibility. On the other hand, FDIs could promote financial sector development which can increase access to clean energy through the availability of funds to support clean energy access. In addition, FDIs increase economic growth and employment, facilitating access to clean energy while the technological transfer associated with FDIs could boost clean energy development. It has been argued that strong institutional controls such as control of corruption can regulate activities of FDIs [21] to promote access to clean energy. However, empirical assessment on this issue is scarce. The moderation effect of corruption control on the women empowerment-access to clean energy nexus is assessed.

Finally, several studies have observed significant associations between economic growth and energy consumption generally, [22–23], and specifically, between economic growth and clean energy consumption [24] while others have examined sectoral growth on carbon emissions or environmental quality [25]. However, the role of sectoral growth on access to clean energy remains largely unexplored. This is relevant because different sectors of the economy utilize energy differently and to different degrees, while the proceeds of growth in one sector may not necessarily translate into increases in clean energy access as compared to examining growth as a composite. Thus, this study makes an addition to the literature by investigating the effect of sectoral growth on access to clean energy.

The remainder of the paper is structured as follows: section 2 reviews the relevant literature, section 3 describes the data and details the methodology adopted to analyze them, section 4 presents the findings and discusses them while section 5 concludes the study.

## 2. Literature review

This section reviews the multifaceted factors influencing access to clean energy in Africa, focusing mainly on the interconnected roles of women’s empowerment, corruption control, FDI, and sectoral growth.

### Women empowerment and clean energy access

Chandrasekaran et al. [26] reported a positive association between the women’s empowerment index and energy access variables, but the association is heterogenous across countries. The study noted that in low and middle-income nations, gender inequality can cause women to bear a disproportionately large burden of energy poverty. The situation is then further perpetuated by limited access to sustainable energy technologies. This is partially due to the fact that women frequently lack autonomy in the home, which probably slows down the adoption of renewable energy technologies by households. This affirms the earlier work of Winthera et al. [27], who viewed women’s empowerment as a progression toward gender parity, encompassing equal rights and access to resources including clean energy. That is to say, women empowerment would potentially increase women’s access to clean energy resources, among others. Opoku et al. [28] confirm that giving women political power could have a positive influence in attaining the SDG targets, including those on clean energy access. Their findings indicate that, the impact of having more women in law making on energy issues such as electricity access, renewable energy consumption, and energy efficiency is influenced by the quality of governance in a country. In the presence of good governance, the effect of having more women in parliament tends to be more pronounced in these energy-related matters. The results were consistent across various scenarios and measurements. This point is further affirmed by DiRienzo and Das [29] who suggest that the presence of women in political positions has a favorable influence on attaining environmental results, however, this influence is manifested via their role in decreasing corruption. The study discovers that a higher proportion of women in political authority is associated with decreased corruption, consequently contributing to an enhancement in environmental performance. Corruption has been noted to negatively impact various macroeconomic aspects, spanning from a nation’s infrastructure quality to its development capabilities. Both Opoku et al. [28] and DiRienzo and Das [29] confirm that giving women the opportunity to have access to political power would have a positive effect towards the attainment of clean energy counteracting the effect of corruption. There are also studies that show the reverse relationship – that access to clean energy could have positive implications for women empowerment [11,30,31]. These studies show that through access to cleaner cooking fuels and the provision of electricity and appliances to women-run businesses, women can be more economically active, more productive at their businesses and ultimately be more empowered.

### Corruption and clean energy access

Corruption significantly affects the overall sustainability of various endeavors, particularly in the context of sustainable energy development. Corruption’s detrimental influence extends across economic, social and environmental spheres, impacting the successful implementation of sustainable energy initiatives. The energy sector, including oil and gas, and power generation, transmission and distribution could be avenues for corruption and indeed are due to the huge rents generated from that sector and the huge role governments play in the value chain (see: http://bpi.transparency.org/bpi2011/). Therefore, addressing corruption is crucial for the effective and sustainable advancement of energy-focused initiatives. Amoah et al. [32] conducted a study on the use of renewable energy in Africa. They argued that in Africa, corruption negatively impacts the use of renewable energy. This results from corrupt officials who take advantage of inefficient bureaucratic systems, remaining hidden as they exploit the weaknesses of public institutions in African economies. Their findings show that corruption has a negative effect on the use of renewable energy with a greater negative effect observed in middle-income African nations than in low-income African nations. Gani [33] also found in a multi-country study that countries with significant energy assets like gas, oil and coal are more likely to experience higher levels of corruption compared to those without such resources. Gennaioli and Tavoni [34] examined the relationship between corruption and public policy for the case of wind energy in Italy. Findings showed that the allocation of public subsidies to support renewable energy initiatives can inadvertently create opportunities for exploitation by criminal elements. It highlights the potential for illicit collaborations to emerge between business leaders and influential political figures who possess the authority to sway the approval of licenses for these energy projects. Consequently, the corruption inhibits significant access to renewable energy since in the presence of weak institutions, even carefully crafted market-focused policies could lead to unfavorable outcomes.

Imam et al. [35] used a panel data of 47 Sub-Saharan African countries from 2002 to 2013 to examine the impact of corruption and two key aspects of electricity sector reforms – the creation of independent regulatory agencies and private sector participation, on several performance indicators in Sub-Saharan Africa. The study found that corruption can make a sector less effective and limit the ability to improve access to electricity, which consequently affects the nation’s income adversely. They also noted that countries located in the Sub-Saharan region of Africa are widely recognized for having some of the most substantial instances of corruption when compared to other countries around the world. Acheampong et al. [36] studied the influence of corruption, income inequality, and redistribution on energy usage across 166 nations. The study found that control of corruption boosts both renewable and non-renewable energy use; and that income redistribution drives total energy consumption as well as renewable energy consumption while decreasing nonrenewable energy use. The study underscores the importance of comprehending the influence of corruption, income inequality, and wealth redistribution on energy consumption, both from renewable and nonrenewable sources. By examining these factors, it becomes possible to develop models that can help guide efforts to reduce greenhouse gas emissions through energy consumption and work towards achieving net zero emissions.

### Sectoral growth and clean energy access

The major sectors of an economy – agricultural, industry, and service sectors – use up energy differently and their respective growth trajectories could have significant implications for access to clean energy. Climate change which is mostly caused by excessive carbon in the atmosphere from conventional energy usage, like fossil fuel, coal and gas can be reduced through the introduction of sustainable energy into industrial operations. Balogun [37] examined the linkage between energy access, renewable energy consumption, and industrial output in Nigeria. He revealed that access to electricity and renewable energy consumption have a positive impact on industrial output, both in the short-run and long-run. Clean energy for cooking boosts industrial output in the short-run while foreign direct investment has a long-run positive impact on industrial output. Indicating that access to clean energy and renewable energy use can increase the industrial sector growth. This could link back to access to clean energy as a result of industrial growth depending then on the type of energy adopted by the firms in the industrial sector. Fotio et al. [38] examines the effect of renewable energy on value-added in the agricultural, industrial and service sectors using data from selected 12 Sub-Saharan African countries from 1985 to 2017. Their findings show that renewable energy significantly improves value-added in these sectors in the long run with the service sector showing the most sustainable increase. Thus, through increasing access to energy and enhancing energy transition, renewable energy is potentially indispensable in achieving global energy targets. Getachew et al. [39] investigated the impact of the sectoral economy on the proportion of renewable energy consumption in Ethiopia. The study found that while the mining, services, and trade sectors of the economy reduced renewable energy consumption, the manufacturing sector had a positive impact on renewable energy consumption. The study further noted that financial assistance in support of green energy was crucial to its consumption. Thus, policies focusing on providing continuous financial support and international collaboration are crucial to maintaining an increasing share of renewable energy consumption in Ethiopia. This could include incentives for adopting renewable energy technologies and investing in renewable energy infrastructure. However, due to the negative impact of service and mining industries on renewable energy use, there is a need to diversify renewable energy sources beyond these sectors. The need for financial support is further amplified by Petrovics et al. [40] who observed that financial incentives are necessary to scale up clean energy use.

### FDI and clean energy access

FDI inflows are an important aspect of many economies and it potentially plays a pivotal role in facilitating clean energy access in developing countries. FDI does not only provide financial assistance to ensure access to clean energy but also provides technological support, which could promote access to renewable energy. Osano and Koine [41] investigated the role of FDI on technology transfer and economic growth in Kenya, focusing on the energy sector in Nairobi. The study showed a positive relationship between FDI and new technology in the energy sector. This has been made possible through technology transfer, which has led to new innovation in production, research, and development. Through the technological advancement brought about by the FDI, economic growth and environmental quality are expected to increase. Dossou et al. [42] examined the relationship between FDI and renewable energy development with a focus on the moderating role of governance quality in 37 sub-Saharan African economies over the period 1996-2020. The study found that FDI positively impacted renewable energy. It was further found that the interaction between FDI and governance quality also had a positive effect on renewable energy, to a greater degree than their respective individual impacts. Sarkodie et al. [43], on the other hand, showed that FDI inflows are associated with increased pollution. This is due to companies in developed countries moving their production into developing countries where it is easier to pollute due to lax regulations, thus confirming the pollution haven hypothesis. In such instances, the companies might end up not adopting renewable energy sources and might actually resort to polluting sources of energy, leading to an adverse relationship between FDI inflows and clean energy consumption. Yousafzai et al. [44], for instance, found in a study of 27 countries, that FDI had a negative and significant effect on clean energy. Thus, while FDI could on one hand, lead to increased consumption of clean energy through its financing and technology transfer potential, it could also lead to increased consumption of heavily polluting fuel especially in developing countries due to reasons advanced by the pollution haven hypothesis.

Overall, the review of the literature suggests that from different contexts and geographical and economic perspectives, corruption and institutional factors, women empowerment, FDI and sectoral growth potentially influence energy access. However, gaps exist in relative to the moderation effects of these factors as well as in respect of the Africa case, which presents a unique opportunity for further examination due to the regions comparably lower rate of access to clean energy. Thus, this study proceeds to investigate the case for Africa to ascertain the direction of impacts of these factors on clean energy access and consequently, on the attainment of SDG 7.

## 3. Methodology

### 3.1. Theoretical framework and empirical modelling

The role of clean energy in the socio-economic development of every economy is well known in literature [45–47]. What has emerged from the literature is the crucial role corruption plays towards this agenda. Corruption leads to huge costs to developing countries especially in Africa, including significant monetary losses. Through such losses the wellbeing of the African, including access to clean energy, is hampered. It stands to reason that if such corrupt practices are controlled it could boost access to clean energy on the continent since more resources could become available to be allocated towards the transition to clean energy. Furthermore, expansion in economic activities which could improve the living condition of individuals has the capacity to increase access to clean energy. However, when economic expansion occurs in ways that do not increase employment avenues, it may not translate into higher access to clean energy. This implies that growth in the agricultural sector, industrial sector and service sector may have different effects depending on the extent to which such growth is associated with welfare enhancement and the proportions of people able to benefit from such growth. In the last two decades women empowerment on the continent has received increasing attention. Such empowerment may not only improve the living conditions of women alone, but those of their immediate families and other dependents. With enhanced women empowerment, women can support their family budgets to acquire clean energy. The empirical analysis controls for the effect of Foreign Direct Investment (FDI). With respect to (FDI), African countries have attracted significant amounts over the years. The effect of such investment on clean environment has been mixed [48–50]. It can equally have mixed effect on access to clean energy. The reason is that FDIs that might have taken advantage of the weak environmental laws on the continent may not be interesting in investing in or utilizing clean energy, but rather take advantage and use polluting energy. On the other hand, when FDIs offer healthy competition, enhances transfer of technological knowledge, and focus on supporting environmentally clean agenda, more clean energy technologies may be produced to enhance accessibility [51,52].

Because the activities of the various economic sectors may be badly affected by corruption related activities it is anticipated that corruption control could moderate the effects of sectoral growth and FDI on access to clean energy. Considering the above arguments and controlling for the effects of mobile technology which could support access to clean energy through innovation and information provision [18,53] and whose development and adoption is growing in Africa; and FDI, which could affect clean energy access either positively or negatively [48–51], our base line model is specified as:


$$\text{ATCE }=\text{f}\left(\text{CCT, WEMP, SECG, MOT, FDI, Y}\right)$$ (1)

where *ATCE, CCT, WEMP, SECG, MOT, FDI,* and *Y* indicate access to clean fuels and technologies, control of corruption, women empowerment, sectoral growth, mobile technology, foreign direct investment, and income respectively. Mobile technology is one of the unconventional variables in access to clean energy models. However, some recent empirical works including [18,53] have accounted for mobile technology in their analyses. They argue that mobile technology promotes clean energy development which could increase availability for people to access. Mobile technology also offers a means to intensify education on clean energy to cause individuals to switch from non-clean energy to clean energy.

Assuming equation (1) is of the nature of a Cobb-Douglass function, it is linearized by taking the natural logarithmic forms of the variables yielding:

$${\text{LATCE}}_{\text{it}}{\text{=α}}_{\text{0}}{\text{+α}}_{\text{1}}{\text{LCCT}}_{\text{it}}{\text{+α}}_{\text{2}}{\text{LFDI}}_{\text{it}}{\text{+α}}_{\text{3}}{\text{LWEMP}}_{\text{it}}{\text{+α}}_{\text{4}}{\text{LMOT}}_{\text{it}}{\text{+α}}_{\text{5}}{\text{LSECG}}_{\text{it}}{\text{+α}}_{\text{6}}{\text{LY}}_{\text{it}}{\text{+ε}}_{\text{it}}$$ (2)

Where α_0_ is the intercept, α_1---_ α_6_ are the coefficients of the explanatory variables, ε contains the error term and country specific effect. *L* denotes the natural logarithm of the variables. Also, *i* and *t* are the individual countries and time dimension of the study respectively.

Following the suggestion that controlling corruption can moderate the effect of sectoral growth, women empowerment as well as FDI inflows, three interactive terms of (LCCT x LSECG), (LCCT x LWEMP) as well as (LCCT x LFDI) are created and added separately to equation (2) to get:

$${\text{LATCE}}_{\text{it}}{\text{=α}}_{\text{0}}{\text{+α}}_{\text{1}}{\text{LCCT}}_{\text{it}}{\text{+α}}_{\text{2}}{\text{LFDI}}_{\text{it}}{\text{+α}}_{\text{3}}{\text{LWEMP}}_{\text{it}}{\text{+α}}_{\text{4}}{\text{LMOT}}_{\text{it}}{\text{+α}}_{\text{5}}{\text{LSECG}}_{\text{it}}{\text{+α}}_{\text{6}}{\text{LY}}_{\text{it}}{\text{+α}}_{\text{7}}\left({\text{LSECG}}_{\text{it}}{\text{x LCCT}}_{\text{it}}\right){\text{ +ε}}_{\text{it}}$$ (3)

$${\text{LATCE}}_{\text{it}}{\text{=α}}_{\text{0}}{\text{+α}}_{\text{1}}{\text{LCCT}}_{\text{it}}{\text{+α}}_{\text{2}}{\text{LFDI}}_{\text{it}}{\text{+α}}_{\text{3}}{\text{LWEMP}}_{\text{it}}{\text{+α}}_{\text{4}}{\text{LMOT}}_{\text{it}}{\text{+α}}_{\text{5}}{\text{LSECG}}_{\text{it}}{\text{+α}}_{\text{6}}{\text{LY}}_{\text{it}}{\text{+α}}_{\text{7}}\left({\text{LFDI}}_{\text{it}}{\text{x LCCT}}_{\text{it}}\right){\text{ +ε}}_{\text{it}}$$ (4)

and

$${\text{LATCE}}_{\text{it}}{\text{=α}}_{\text{0}}{\text{+α}}_{\text{1}}{\text{LCCT}}_{\text{it}}{\text{+α}}_{\text{2}}{\text{LFDI}}_{\text{it}}{\text{+α}}_{\text{3}}{\text{LWEMP}}_{\text{it}}{\text{+α}}_{\text{4}}{\text{LMOT}}_{\text{it}}{\text{+α}}_{\text{5}}{\text{LSECG}}_{\text{it}}{\text{+α}}_{\text{6}}{\text{LY}}_{\text{it}}{\text{+α}}_{\text{7}}\left({\text{LWEMP}}_{\text{it}}{\text{x LCCT}}_{\text{it}}\right){\text{ +ε}}_{\text{it}}$$ (5)

In every economy the key sectors are the agricultural sector (AGS), industrial sector (IND) and services sector (SERS). The log values of the three sectors’ growths – agricultural sector (LAGS), industrial sector (LIND), and service sector (LSERS) – will separately replace sectoral growth (SECG) in equations 4 and 5. In addition because the natural log of FDI and CCT takes on non-positive integers the final estimations did not use the log terms for those two variables.

### 3.2. Data and estimation techniques

The study relies on data from 32 African countries with sufficient data for the variables of interest. The analyses cover the period of 2002–2021 and this is informed by data availability. Table 1 shows the description, measurement and source of each variable.

**Table 1:** Data description, measurement and source.

Variable	Description	Measurement	Source
LY	Income	Log of GDP	World Bank 2023
WEMP	Women empowerment	Log of women, business and law index. The index measures how laws and regulations affect women’s economic opportunity. Overall scores are calculated by taking the average score of each index (Mobility, Workplace, Pay, Marriage, Parenthood, Entrepreneurship, Assets and Pension), with 100 representing the highest possible score.	World Bank 2023
CCT	Control of corruption	Control of corruption index	World Bank 2023
FDI	Foreign Direct Investment	Net FDI (% GDP)	World Bank 2023
MOT	Mobile phone subscription	Log of number of people using mobile phones	World Bank 2023
AGS	Agric sector growth	Log of agricultural value addition	World Bank 2023
IND	Industrial sector growth	Log of industrial value addition	World Bank 2023
SERS	Service sector growth	Log of service value addition	World Bank 2023

Table 2 shows the descriptive statistics and correlation of the variables to get an initial picture of the data used for the analysis.

**Table 2:** Descriptive statistics and correlation.

Statistics	ATCE	WEMP	CCT	FDI__	MOT	SERS	IND	AGS
Mean	28.96813	63.48651	−0.620455	4.373853	16072313	46.89514	25.34781	20.51695
Median	9.250000	63.75000	−0.706012	2.291233	5690751.	47.58295	24.01534	20.18792
Maximum	99.90000	89.37500	1.244920	103.3374	2.04E + 08	68.02736	66.17917	79.04236
Minimum	0.100000	28.12500	−1.581135	−11.19898	0.000000	17.86371	3.243096	1.738935
Std. Dev.	35.47343	14.07991	0.569233	8.853195	26644932	8.751399	11.37012	12.92468
Correlation	ATCE	WEMP	CCT	FDI__	MOT	SERS	IND	AGS
ATCE	1.000000							
WEMP	0.007805	1.000000						
CCT	0.327391	0.282865	1.000000					
FDI	−0.138639	−0.023101	−0.024905	1.000000				
MOT	0.246577	0.107072	−0.183728	−0.123906	1.000000			
SERS	0.414556	0.292559	0.363980	−0.165133	0.200006	1.000000		
IND	0.342525	−0.207880	−0.066474	−0.053134	0.103025	−0.239199	1.000000	
AGS	−0.651748	−0.143062	−0.263337	0.203897	−0.192592	−0.479916	−0.644180	1.000000

Panel data are often associated with cross sectional dependence due to globalization and this can affect the type of test used to examine the stationarity properties of the variables which also have implications for the results from regression analysis. As a result, the study first of all assessed the nature of cross-sectional dependence of the variables using the Breusch-Pagan LM, Pesaran scaled LM and Pesaran CD test. After this the unit root test is conducted. The unit root test is carried out to check the stationarity properties of the variables. Non-stationary variables in estimating regression models lead to spurious estimates and consequently, wrong conclusions. We therefore use the CIPS unit root test, determined to be appropriate to test for stationarity, following the result of the CD test result. Furthermore, it has been documented that panel data tend to have a long run relationship among the variables. Such a situation implies a cointegration relationship. This study used the Pedroni test to assess the relationship. After a confirmation of cointegration, the effect of income, women empowerment, corruption control, FDI, mobile technology and sectoral growth on access to clean energy is explored with the fully modified ordinary least squares (FMOLS). This method is appropriate since it addresses the problem of endogeneity and serial correlation often associated with panel data. It is given by:

$${\hat{\beta }}_{fmol}={\left[\sum_{i=1}^{N}\sum_{t=1}^{T}(x_{it}-{\bar{x}}_{i}{)}^{'}\right]}^{-1}\left[\sum_{i=1}^{N}(\sum_{t=1}^{T}(x_{it}-{\bar{x}}_{i}){\hat{y}}_{it}^{+}+T{\hat{\Delta }}_{\varepsilon \mu }^{+}\right]$$ (6)

where ${\hat{\Delta }}_{\varepsilon \mu }^{+}$ is the serial correlation correction term and ${\hat{y}}_{it}^{+}$ is the transformed variable of *y*_*it*_ to achieve the endogeneity correction.

## 4. Results and discussion

This section presents the results of the study. from initial diagnostic tests on cross-sectional dependence, stationarity and cointegration. These are followed by the FMOLS regression estimates for the baseline model as well as the models that examine moderating effects.

Table 3 reports the results of the Pesaran scaled LM and the Pesaran CD tests. The results indicate the presence of cross-sectional dependence in the residuals of the model. The test statistics are highly significant and this shows strong evidence against the null hypothesis of no cross-sectional dependence. Hence, the CIPS test is used to test for stationarity of the variables. The Breush Pagan test also suggests the presence of heteroscedasticity in the residuals.

**Table 3:** Cross-sectional dependence of residuals.

Test	(1)	(2)	(3)
Breusch-Pagan LM	2709.846***	2357.464***	2747.968***
Pesaran scaled LM	70.28968***	59.10153***	71.50005***
Pesaran CD	10.47286***	3.282516***	14.09493***

***p < 0.01; (1) - Model with agricultural growth; (2) - Model with industry growth; (3) - Model with service growth.

Source: Authors’ construction

### Test for Stationarity of Variables

Table 4 presents the CIPS unit root test of the variables. The results indicate that all the variables are non-stationary at levels but stationary at first difference, hence the variables are appropriate for regression estimations with the assurance that these regressions will not generate spurious results.

**Table 4:** CIPS Unit root test results.

Variable	At levels	First difference
LY	−2.09	−3.49***
LWEMP	1.06	−13.754***
CCT	−2.18	−4.37***
LATCE	−2.04	−4.84***
FDI	−0.54	−7.36***
LMOT	−0.717	−3.47***
LAGS	−2.19	−3.47***
LIND	−2.70	−3.42***
LSERS	−2.20	−3.60***

*** p < 0.01.

### Panel cointegration

Having established that all the variables are integrated of order one, it is necessary to investigate the relationship among the variables in the long run. The cointegration test is carried out to ascertain whether a stable equilibrium relationship exists between the dependent variable and the explanatory variables in the long run. The test is necessary because even if variables in a model do not satisfy stationarity properties, their linear combination in a model could be stationary. The results are reported in Table 5. In all models, the within-dimension tests (Panel v, Panel PP, Panel ADF) provide consistent evidence of cointegration. Similarly, the between-dimension tests (Group PP) also suggest strong evidence of cointegration. The results overall suggest the presence of cointegration among the specified variables, indicating a long-term relationship. We therefore proceed to estimate the model to know the effect of women empowerment, corruption control and sectoral growth on access to clean energy while accounting for relevant covariates. The baseline results are presented in Table 6.

**Table 5:** Panel cointegration results.

	(1)		(2)		(3)	
Test	Statistic	Weighted Statistic	Statistic	Weighted Statistic	Statistic	Weighted Statistic
Panel v-Statistic	2.623***	3.626***	3.705***	2.087**	2.594***	3.507***
Panel rho-Statistic	5.367	4.303	5.646	4.679	5.304	4.327
Panel PP-Statistic	−6.941***	−7.941***	−4.733***	−6.775**	−7.232***	−7.646***
Panel ADF-Statistic	−5.081***	1.099	−4.616***	2.496	−4.456***	1.477
Group rho-Statistic	5.953		6.155		6.147	
Group PP-Statistic	−9.066***		−10.048***		−10.467***	
Group ADF-Statistic	1.903		1.993		1.743	

*** p < 0.01 **p < 0.05; (1) - Model with agricultural growth; (2) - Model with industry growth; (3) - Model with service growth.

**Table 6:** Base line estimation results.

	(1)		(2)		(3)	
Variable	Coefficient	Std. Error	Coefficient	Std. Error	Coefficient	Std. Error
LY	0.699***	0.024	0.655***	0.022	0.664***	0.021
LWEMP	0.429***	0.029	0.401***	0.028	0.308***	0.027
CCT	0.353***	0.014	0.302***	0.015	0.388***	0.013
FDI	−0.001***	0.0003	−0.001***	0.0003	−0.001***	0.0003
LMOT	0.037***	0.005	0.055***	0.004	0.050***	0.004
LAGS	0.078***	0.018				
LIND			−0.152***	0.015		
LSERS					−0.537***	0.025
Adj-R^2^	0.97		0.97		0.97	

*** p < 0.01; (1) - Model with agricultural growth; (2) - Model with industry growth; (3) - Model with service growth.

All coefficients are statistically significant at the 1% level, indicating a high level of confidence in the results. The results also suggest strong associations between the independent variables and the dependent variable, with high explanatory power as indicated by the adjusted R-squared value of 97% in all three models. In all models, women empowerment and corruption control have positive and statistically significant effects on access to clean energy while the impact from sectoral growth differs by sector. A 1% improvement in the level of women empowerment leads to between a 0.31% − 0.43% increase in access to clean fuels and technologies in Africa. At the household level, women are often the ones assigned the cultural role of preparing family meals. This implies that they are the ones who have direct contact with the household cooking energy. Thus, as they become empowered, they are likely to opt for cleaner cooking fuels and technologies which benefit them by saving them time while keeping them from suffering the adverse health effects of using heavily polluting fuel. Furthermore, technologies, such as efficient cookstoves that enhance the efficiency of fuel use will be of interest to women since it helps the save on the budget for running the home. In addition, women empowerment reduces the burden of poverty borne by women. As such women are able to contribute financially to support clean energy purchases at the household level and for their businesses as well. They would therefore opt for those when they are empowered. This agrees with the findings of [26,28].

An increase in corruption control was also found to have a positive effect on access to clean energy. Corruption often deprives an economy the needed funds to pursue issues of development interests by channeling public funds into private pockets. The development targets that could be impaired include extending access to clean energy and efforts to support the uptake of clean fuels and technologies. Thus, controlling corruption increases funds available for promoting access and uptake of clean energy. This is consistent with previous studies that found that corruption limits renewable energy consumption in Africa [32,35]. In column (1) of Table 6, it is observed that agricultural sector growth is positively associated with access to clean energy in Africa. Agriculture is a mainstay for many economies in Africa with large proportions of rural households engaged in this sector while the sector by itself is not as heavily reliant on energy compared to the industrial and service sectors. The implication is that growth in the sector is likely to lead to increased incomes for the economic agents engaged in that sector. The increased incomes could then be used to access clean energy and technologies, leading to the observed positive relationship between agricultural sector growth and access to clean energy. In the case of the industrial and service sectors, a negative relationship between the respective sectors and access to clean energy was observed. This is likely the case if the industrial sector, which relies heavily on energy for its operations, use traditional and heavily polluting energy sources. As long as the energy that is used as inputs in the industrial sector are not from modern and cleaner sources, growth in the sector will invariably imply growth in the use of dirty energy sources. This supports the findings of Getachew et al. [39] who found that the mining sector, which is a key component of the industrial sector had a negative relationship with renewable energy consumption in Ethiopia. Similar argument could be made for the service sector growth’s negative association with access to clean energy. The sector might be relying more on traditional sources of energy thus, as the sector grows, more of such energy is consumed leading to the negative relationship with clean energy access.

As expected, income has a positive impact on clean energy access and agrees with the findings of [7,18,54]. Increased income makes it possible to afford clean energy, while several studies have shown that as an economy’s income increases, they tend to demand cleaner environment and therefore likely to pursue programs that improve environmental quality, which would include programs that enhance use of clean energy and technologies. Mobile subscriptions also showed a positive effect on access to clean energy. A 1% increase in mobile telecommunication subscriptions leads to 0.04%–0.06% increase in access to clean energy. Access to mobile technology in this age increases the information flow to people. Thus, they are able to get information about clean energy and its benefits, the dangers of using polluting energy and the value of a clean environment generally. This increases their likelihoods of choosing cleaner energy and technologies. This also confirm findings from micro studies that have shown that access to information and awareness about the environment, which may be obtained through access the use of mobile phones, influence clean energy choices [55,56]. The findings, however, contradict those of Wang et al. [57] who found that information communication technology (ICT) had a limited effect on reducing energy poverty among vulnerable groups and intensifies energy poverty for those already experiencing the phenomenon. While mobile technology might be closely associated with ICT, the two might differ quite significantly within the context of our study area. This could be responsible for the contrasting results of [57]. FDI, on the other hand, has a negative influence on access to clean energy. An increase in FDI inflows reduces access to clean energy holding other variables constant. This might appear counter-intuitive at first instance and actually contrasts the findings of previous literature [7]. However, the finding is not ridiculous. A reasonable explanation is that if the FDI inflows into Africa come to support industries and economic activities that rely on heavily polluting energy sources and not to build the renewable and clean energy sector, then it is expected that increases in FDI inflows would have a negative relationship with access to clean energy.

### Moderation analysis

The results for moderation analyses conducted for different sets of variables are presented below. Table 7 presents the results of the moderation analyses for corruption control and sectoral growth. It was found that controlling corruption further reinforced agricultural sector growth’s positive effect on access to clean energy. In the case of the industrial sector, controlling corruption reverses the initial negative effect of growth of the sector on access to clean energy while in the case of the services sector, controlling corruption reinforces the negative effect of the sector’s growth on access to clean energy. This implies that the effect of corruption control within the services sector is not strong enough to reverse the negative impact of the sector’s growth on access to clean energy whereas in the case of the industrial sector, corruption control is able to reverse the negative impact of the sector’s growth on access to clean energy. However, it is still not fruitless to embark on corruption control measures since corruption control reduces the magnitude of the negative effect of service sector growth on access to clean energy. It can be seen from the baseline results that the magnitude of the effect of service sector growth on access to clean energy is nearly four times as much as that of the industrial sector growth’s effect on access to clean energy. Given the stronger effect in respect of the service sector, it would require greater corruption control efforts and better results for that effect to be reversed. It is also important to note that corruption control is actually low in the study region (Africa) with a negative mean value (see: Table 2). This is expected since corruption is high in most of the public service [58] which also controls the activities of most of the service sub-sectors including transport, storage, and communications, restaurants and hotels, and insurance in Africa. Thus, the extent of corruption control is still low and that, together with the strong negative effect originally observed in respect of the service sector growth reasonably explains the inability of corruption control to reverse the negative effect of the service sector growth on access to clean energy.

**Table 7:** Results from moderation analysis for corruption control and sectoral growth.

	(1)		(2)		(3)	
Variable	Coefficient	Std. Error	Coefficient	Std. Error	Coefficient	Std. Error
LY	0.658***	0.0052	0.669***	0.019	0.628***	0.005
LWEMP	0.470***	0.014	0.426***	0.025	0.328***	0.011
CCT	−0.507***	0.012	−0.828***	0.067	0.665***	0.014
FDI	0.030	0.020	−0.001***	0.0003	0.013	0.020
LMOB	0.051***	0.007	0.055***	0.004	0.048***	0.007
LAGS	0.271***	0.007				
LAGS x CCT	0.306***	0.009				
LIND			0.092***	0.019		
LIND x CCT			0.379***	0.022		
LSERS					−0.492***	0.014
LSERS x CCT					−0.070***	0.009
Adj-R^2^	0.96		0.97		0.97	

*** p < 0.01; (1) - Model with agricultural growth; (2) - Model with industry growth; (3) - Model with service growth.

Table 8 shows the results of the moderating effect of corruption on the relationship between FDI and access to clean energy. It is observed that corruption control reinforces the negative effect of FDI on access to clean energy in all models. This simply implies that increase in FDI inflows tend to reduce clean energy access and control of corruption does not reverse the negative effect. As explained earlier, corruption control is at low levels in the study region and therefore in some cases, is unable to yield the expected impact especially when interacting with other established factors at play in the economy. The implication is for more active efforts at controlling corruption in order for it to be forceful enough to permeate other variables in the economy to yield significant gains.

**Table 8:** Results from moderation analysis for corruption control and FDI.

	(1)		(2)		(3)	
Variable	Coefficient	Std. Error	Coefficient	Std. Error	Coefficient	Std. Error
LY	0.671***	0.019	0.638***	0.019	0.644***	0.016
LWEMP	0.414***	0.023	0.391***	0.024	0.299***	0.020
CCT	0.457***	0.013	0.402***	0.014	0.484***	0.011
FDI	−0.016***	0.001	−0.015***	0.0008	−0.014***	0.0007
FDI x CCT	−0.021***	0.001				
FDI x CCT			−0.020***	0.001		
FDI x CCT					−0.020***	0.0009
LMOT	0.047***	0.004	0.061***	0.004	0.057***	0.003
LAGS	0.085***	0.014				
LIND			−0.147***	0.012		
LSERS					−0.519***	0.019
Adj-R^2^	0.97		0.97		0.97	

*** p < 0.01; (1) - Model with agricultural growth; (2) - Model with industry growth; (3) - Model with service growth.

From Table 9 where the moderation analysis of corruption control on the effect of women empowerment it is observed that corruption control reduces the positive effect women empowerment has on access to clean energy. This outcome may be due to the fact that generally corruption control in Africa is weak and the little done is unable to support women empowerment in promoting access to clean energy.

**Table 9:** Results from moderation analysis for corruption control and women empowerment.

	Agricultural Model		Industry Model		Service Model	
Variable	Coefficient	Std. Error	Coefficient	Std. Error	Coefficient	Std. Error
LY	0.776***	0.021	0.728***	0.018	0.734***	0.016
LWEMP	0.129***	0.031	0.090***	0.029	0.117***	0.026
CCT	1.525***	0.118	1.544***	0.108	0.978***	0.098
FDI	−0.001***	0.0003	−0.001***	0.0002	−0.0009***	0.0002
LMOT	0.043****	0.004	0.061***	0.003	0.055***	0.003
LAGS	0.108***	0.015				
LIND			−0.178***	0.012		
LSERS					−0.577***	0.019
LWEMP x CCT	−0.267***	0.028	−0.284***	0.025	−0.127***	0.023
Adj-R^2^	0.97		0.97		0.97	

***P  <  0.01.

## 5. Conclusion

Climate change and its associated effects on the natural environment and the health of people is a major global concern. This is especially the case for developing countries, which have a lower capacity to contain the menace and reduce the negative impacts on its people. Among the factors that can be tackled to address the situation, energy consumption is key. This is because of the major role it plays in contributing to global warming and climate change as a key input into production at both the micro and macro levels. The attainment of SDG goal 7 – accessibility of clean, modern and sustainable energy for all can therefore be a crucial step in slowing down the rate of climate change and consequently, mitigating its impact in Africa. To this end, this study revisited the subject of access to clean energy in Africa, investigating the roles of women empowerment, corruption control and sectoral growth. Data from a panel of 32 African countries over a 20-year period (2002–2021), estimated using the FMOLS technique shows that women empowerment, controlling corruption and agricultural sector growth increases access to clean energy while growth in the industrial and service sectors have an adverse effect on access to clean energy. The evidence further showed that mobile telecommunication subscriptions and income also have positive effects on access to clean energy while FDI tends to have a negative effect on clean energy accessibility. Further analyses showed that corruption control is able to reinforce the effect of agricultural sector growth and reverse the negative effect of industrial sector growth while reinforcing the negative effects of service sector growth and FDI on access to clean energy.

On the bases of the foregoing, the study recommends that measures that enhance women empowerment be pursued by the economies of Africa. More programs that target female education, female economic and social opportunities, female decision-making and reduction in gender inequality on all fronts should be embarked upon and funded by governments in Africa. This will be significant in achieving increased access to clean energy. The findings of the study show that corruption control is still at low levels in Africa and that corruption control should exist at high levels in order for it to have a positive moderating effect on other variables. Therefore, existing corruption control measures need to be strengthened and the scope widened to achieve higher corruption control indices across the continent. This could be done through measures including but not limited to resourcing anti-graft bodies and economic crime investigation agencies, giving these institutions the required independence to operate and pursuing significant attitudinal changes in respect of societal values that eschew corruption. Finally, a balanced sectoral growth is recommended. Pursuing industrialization or growth in the service sector without neglecting the agricultural sector is important. This is because the agricultural sector’s growth effect on access to clean energy is significant and with a huge proportion of workers in this sector in Africa, growth in the sector inures to the economic benefit at both the micro and macro levels, permitting wider adoption of clean fuels and technologies. Furthermore, policies that incentivize the widespread adoption of cleaner energy in the industry and service sectors are recommended. For example, financial incentives from governments and multilateral agencies and fiscal support from governments including tax incentives or related subsidies to adopt renewable forms of energy for actors in these sectors is crucial. This will ensure that growth in these sectors will be accompanied by growth in cleaner energy use. Of course, pursuing overall economic growth with distributed benefits for all is critical to promoting access to clean energy.

This study is limited by the inability to empirically test some mechanisms through which the factors found to be significant might affect access to clean energy due to data limitations. A future study that does this will contribute to advancing the frontiers of knowledge on the subject matter. Furthermore, this study stops short of testing for error correction due to missing data. Furthermore, data limitations do not allow us to assess the effects on the various sub-sectors of the service sector vis-à-vis the peculiar corruption control regimes/mechanisms that prevail. Another limitation owing to data constraints is the inability of the current study to examine how different types of FDI inflows influences clean energy. It is therefore recommended that future studies address these when sufficient data is available.

## Supporting information

S1 Table Descriptive and correlation of variables in logs. (DOCX)
