# Supplementary material for: Access to clean energy in Africa revisited: The roles of women empowerment, corruption control, FDI and sectoral growth
Source: PLoS One. 2025 Feb 4;20(2):e0317781. doi: 10.1371/journal.pone.0317781 (PMC11793778; doi:10.1371/journal.pone.0317781)
Supplement: S1 Table — (DOCX) [file pone.0317781.s001.docx]

S1 Table. Descriptive and correlation of variables in logs.

| Statistics | LATCE | FDI | CCT | LAGS | LMOT | LIND | LSERS | LWEMP |
| --- | --- | --- | --- | --- | --- | --- | --- | --- |
| Mean | 2.145713 | 4.387048 | -0.619646 | 2.766964 | 15.36348 | 3.134762 | 3.829169 | 4.125409 |
| Median | 2.251292 | 2.301949 | -0.706012 | 2.999236 | 15.55735 | 3.179972 | 3.862475 | 4.154969 |
| Maximum | 4.604170 | 103.3374 | 1.244920 | 4.369984 | 19.13475 | 4.192366 | 4.219910 | 4.492841 |
| Minimum | -2.302585 | -11.19898 | -1.581135 | 0.553273 | 7.150701 | 1.176528 | 2.882771 | 3.336659 |
| Std. Dev. | 1.854437 | 8.865245 | 0.569925 | 0.804121 | 1.868902 | 0.462744 | 0.198599 | 0.236317 |
|  |  |  |  |  |  |  |  |  |
| Correlation | LATCE | FDI | CCT | LAGS | LMOT | LIND | LSERS | LWEMP |
| LATCE | 1.000000 |  |  |  |  |  |  |  |
| FDI | -0.179805 | 1.000000 |  |  |  |  |  |  |
| CCT | 0.286417 | -0.025506 | 1.000000 |  |  |  |  |  |
| LAGS | -0.741565 | 0.138181 | -0.301498 | 1.000000 |  |  |  |  |
| LMOT | 0.331660 | -0.075361 | -0.118319 | -0.204054 | 1.000000 |  |  |  |
| LIND | 0.520113 | -0.140339 | 0.009249 | -0.611587 | 0.323862 | 1.000000 |  |  |
| LSERS | 0.346364 | -0.166208 | 0.360542 | -0.410008 | 0.154335 | -0.050291 | 1.000000 |  |
| LWEMP | 0.064174 | -0.019683 | 0.279463 | -0.150492 | 0.317387 | -0.136987 | 0.259853 | 1.000000 |
